# Guidelines in Designing a Universal Primer Mixture to Probe and Quantify Antibiotic-Resistant Genes Using the Polymerase Chain Reaction (PCR)

**DOI:** 10.7759/cureus.69479

**Published:** 2024-09-15

**Authors:** Andrew Hui

**Affiliations:** 1 Department of Biological Sciences, University of Calgary, Calgary, CAN

**Keywords:** anti-biofilm, antimicrobial resistance by biofilms, diagnostic, polymerase chain reaction (pcr), primer

## Abstract

Multidrug resistance efflux pumps (MDREPs) in biofilm communities have become an increasingly expensive problem in clinical settings. Polymerase chain reaction (PCR)-based detection can be used to diagnose and characterize these genes, but this requires effective primer design to minimize false positives and negatives in test conclusions. A universal primer approach has previously been used to detect conserved core genes but not for accessory genes such as MDREPs. This study describes a guideline for the design of primers used in the detection of MDREP genes and an optimization approach for creating primers by using multiple sequence alignments to target conserved regions *in silico*, progressing from *in silico* to *in vitro* to generate working primers. Using this approach, this paper was able to generate primers to target *sugE*, a small multidrug resistance (SMR) protein found in microbial species. Primers were tested positively against synthetic DNA sequences but were inconsistent with DNA extracted from the organism of interest. Primer design informs the shortfalls of this detection technique and the difficulty in characterizing such genomic elements.

## Introduction

A biofilm begins to form when microbial cells irreversibly attach to surfaces. This initiates a cellular process where a physical three-dimensional scaffold is formed through the secretion of exopolysaccharides, extracellular DNA, lipids, and proteins [1]. Biofilm-associated infections are common in clinical care and exhibit tolerance to antibiotics [2]. Large-sized and charged compounds are tolerated due to the diffusion barrier created by the biofilm [3,4], while sublethal doses of biocides have been shown to upregulate multidrug resistance pumps (MDREPs) allowing the persistence of these microbial communities [5].

Furthermore, a small number of dormant cells called persisters that aren't removed by an antibiotic treatment can re-establish the community [6]. Multidrug resistance pumps are a vast range of non-conserved integral membrane proteins known for their ability to confer resistance to antibiotics [7,8]. These proteins are divided into six superfamilies: ATP-binding cassette (ABC), major facilitator superfamily (MFS), multidrug and toxic extrusion (MATE), resistance-nodulation-cell division (RND), small multidrug resistance (SMR), and proteobacterial antimicrobial compound efflux (PACE) [5]. The term "multidrug" refers to the ability to efflux multiple substrates that do not share similar structural, size, or ionic properties [9-11]. The focus on MDREPs is due to the fact they are found on mobile genetic elements allowing for horizontal mobility across microbial communities [12]. sugE in particular is an SMR that is involved in the resistance of quaternary ammonium compounds (QACs) [13]. The tightly packed environment of a biofilm makes an ideal environment for this horizontal mobility to occur compared to microbial cells in the aqueous phase [14]. Concerns about the lack of critical information on the transfer of resistance genes within a community bring the need for the evaluation of susceptibility of bacterial populations to treatments in clinical settings [15]. High-throughput sequencing has been successfully used for environmental samples of biofilms [16]. However, primers used in sequencing typically target conserved genes, with the most successful example being the targeting of the 16S rRNA gene, due to its low rate of mutation over time [17].

For an accurate and effective assessment, effective primer design is crucial to prevent a false positive or negative result. Hence, most primers are designed to detect conserved regions for the organism of interest to provide a reliable method of diagnosis [18]. However, such approaches reduce the usability of such primers by limiting the depth of diversity that can be detected [19]. The “universal” primer approach solves this issue by designing a mixed set of primers with differing base variability at defined positions to broadly target specific genetic elements across species [18,19]. The most successful attempt is in targeting the 16S rRNA gene due to its low mutability over time [17]. Other molecular biology techniques such as fluorescence in situ hybridization (FISH), Southern blots, and microarrays also utilize primer-dependent assays and would benefit from successful primer development [20].

This study explores the development of low-cost “universal” PCR primers for the detection of MDREP genes. Unlike the approach taken by 16S designs, MDREP families have little conservation within superfamilies limited to specific residues for function elements or short motifs [21,22], and there are no known conserved elements across superfamilies. This work utilized primer design in silico to target gene-specific conserved regions using multiple sequence alignments (MSAs), which can be used to detect the presence of MDREPs in microbial communities.

## Materials and methods

Gene-mining antibiotic resistance gene identification

A previously established six-bacteria mixed species was used to represent a simplified biofilm community [18]. The chosen strains with their IMG Genome ID numbers are as follows: *Acetobacterium woodii *(2512047040), *Bacillus subtilis *(2511231064), *Desulfovibrio vulgaris *(637000096), *Geoalkalibacter subterraneus *(2593339207), *Pseudomonas putida *(641522645), and *Thauera aromatica *(2791355019).

Using MEGARes, a database of published antibiotic resistance gene sequences for antimicrobial drugs, biocides, and metals [23]. Gene targets were screened based on biocide efflux substrate through keyword identification. Examples of abbreviations or keywords include multidrug, efflux, transporter, outer membrane, inner membrane, and resistance. Genes were chosen with at least one gene in the model community and were used as targets. 

sugE sequence collection and multiple sequence alignment

The gene sugE from the small multidrug resistance (SMR) efflux pump family was selected and the full coding region of sugE nucleotide sequences was retrieved from the NCBI database (https://www.ncbi.nlm.nih.gov/). Sequences were selected to give a range of organisms among different genera (*Aeromonas molluscorum*, *Pseudomonas fluorescens*, *Klebsiella pneumoniae*, *Escherichia coli*, *Sphingomonas*, and *Clostridium kluyveri*), in addition to the sequences in the model community (*Pseudomonas putida*), for a total of seven gene sequences, were imported into the cloud-based informatics platform Benchling (https://www.benchling.com/).

Sequences were aligned using the MAFFT algorithm parameters used in a previous study (max refinement iterations: 0; tree rebuilding: 2; gap open penalty: 1.53; gap extension penalty: 0.0; adjust direction: false) to generate a multiple sequence alignment (MSA) [24].

Primer design

Sequence identity in the alignment was examined for conserved regions for amplicon primer design. Local regions of high sequence identity were targeted with primer designs to reduce the use of degenerate bases to account for mismatches. The total percent sequence identity was calculated using the equation [18]:

Percent identity = (matches * 100) / length of MSA

The 150-nucleotide upstream region and the 250-nucleotide downstream region were targeted due to the consensus sequence identity. Matching the most closely conserved sequences into smaller subsets of groups, three different primer sets, sugE1, sugE2, and sugE3 were designed for the three subgroups of the alignment that are the closest related to each other. Primer sets were synthesized by IDT (Canada) and Eurofin (Canada).

In silico primer targeting of community bacteria

Full-genome sequences of mixed-species community were obtained using the NCBI and imported into a web-hosted sequence management tool (Benchling) for sequence analysis. Primer designs were screened on microbial genomes to detect unannotated sugE sequences. Successful binding of primers on unannotated parts of the genome would indicate the presence of sugE. 

Primer testing through PCR

The accuracy of the primer set was verified through PCR amplification of the positive control and a synthetic piece of double-stranded DNA fragments (gBlock) was used to optimize PCR conditions (Integrated DNA Technologies, IDT). PCR reagents were obtained from ThermoFisher using the following conditions: 2X PCR Master Mix, forward and reverse primers at 0.1 mM, and a template. Cycle parameters were 95 °C for three minutes, followed by 35 cycles of 95 °C for 45 seconds, at five degrees less than the melting temperature for 60 seconds, and 72 °C for 90 seconds, with a final extension at 72 °C for 10 minutes run on the C1000 Touch Thermal Cycler (BioRad). Parameters were chosen based in reference to successful 16S rRNA temperatures and times [18]. Gradient PCR was performed with an increasing annealing temperature based on the melting temperature of each primer set. A premixed master mix was used to minimize side effects from contamination (Thermo Fisher Scientific, USA). The optimal primer concentration was determined using matrix PCR.

Verification of the genomic DNA quality

To confirm working genomic DNA, a 16S primer set was used to verify the appropriate extraction of genomic DNA by creating a baseline. In this PCR protocol, 2 uL of 23.2 ng/uL of DNA was used as a template under the following PCR cycling conditions: 94 °C for three minutes, 35 cycles of 94 °C for 30 seconds, 50 °C for 40 seconds, and 72 °C for one minute followed with a final elongation step at 72 °C for 10 minutes. The amplified product was visualized using a 1.5% agarose gel at 100 V for 50 minutes stained with Gel Red nucleic acid staining solution (Biorad) for 15 minutes. A positive band would be constituted by a 16S rRNA amplification product at approximately 1500 bp. 

DNA extraction for amplification

DNA was collected from bacterial cells using FastDNA Spin DNA Kit (MP Biomedicals, USA). DNA was cleaned using the OneStep PCR Inhibitor Removal Kit (Zymo Research, USA). The concentration of DNA was measured using a Qubit4 fluorometer (Invitrogen, USA).

## Results

In silico design

This study describes a guideline for developing primers to target antibiotic resistance genes. To start, an antibiotic-resistant gene of interest must be selected to develop a multiple sequence alignment. The optimal target sequence is one where most nucleotides are conserved across species. In designing primers, a balance between sensitivity and specificity exists, where the overuse of degenerate nucleotides reduces the specificity to the intended target leading to nonspecific annealing, while underuse reduces the sensitivity to the intended target across organisms. Benchling is a web-hosted DNA sequence manipulation tool that allows visualization of the intended target while considering the melting temperature (Tm), amplicon size, and primer length. These primers can be then validated in silico to confirm proper binding and potential non-intended sites can be identified with changes in stringency parameters. This can be confirmed experimentally by optimizing the annealing temperature using gradient PCR, taking the melting temperature as the maximum annealing temperature, testing down to T-10, and identifying the temperature with the highest amplification. The concentration of the primer set can be optimized with matrix PCR; by testing differing combinations of primer sets, identification of the highest amplification and the absence of primer dimers is important to minimize downstream problems.

**Figure 1:**
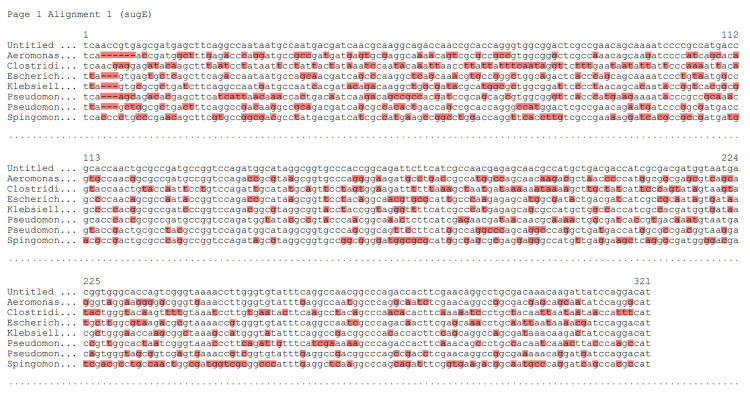
Multiple sequence alignment of sugE isoforms. Organisms used were Aeromonas molluscorum (gram-negative proteobacteria), Pseudomonas fluorescens (gram-negative proteobacteria), Klebsiella pneumoniae (gram-negative proteobacteria), Escherichia coli (gram-negative proteobacteria), Sphingomonas (gram-negative proteobacteria), and Clostridium kluyveri (gram-positive firmicutes). Unhighlighted regions are conserved, while red-highlighted denotes regions that are not.

The design was based on a previously established model microbial community to probe the genomes of organisms for potential antibiotic resistance genes. The identified target was sugE, a member of the small multidrug-resistant (SMR) family, which can efflux a multitude of biocides, including quaternary ammonium compounds (QACs). Seven isoforms of sugE were picked to create the multiple sequence alignment for primer design to design a “universal” mixture (Figure 1).

**Figure 2:**
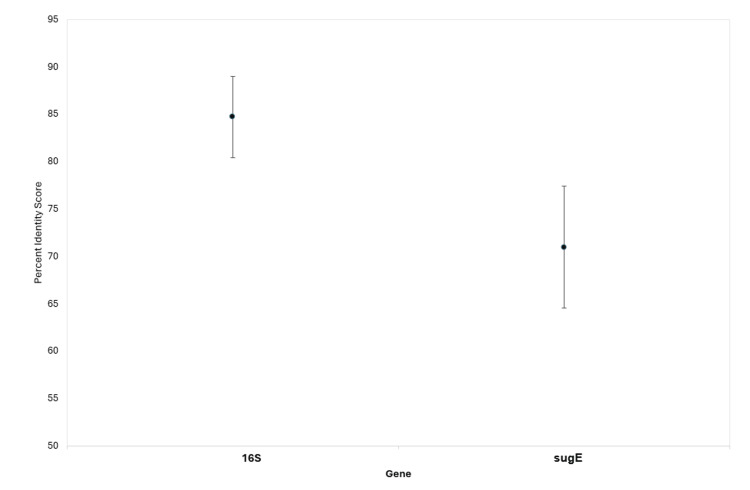
Average percent identity score of genes used in the study. Scores were calculated using the MSA length and the number of matches. The plot shows the average percent identity while the bars represent the SEM. 16S MSA length was 1595 bp made from 32 different genes, giving a percent identity score of 84.71 ± 4.32. The sugE MSA length was 321 bp made from seven different genes, giving a percent identity score of 70.98 ± 6.43. MSA: multiple sequence alignment

To provide an idea of the conservation of sugE across the species, sequence identity was calculated and found using a t-test to be significantly lower than that of 16S (Figure 2).

**Figure 3:**
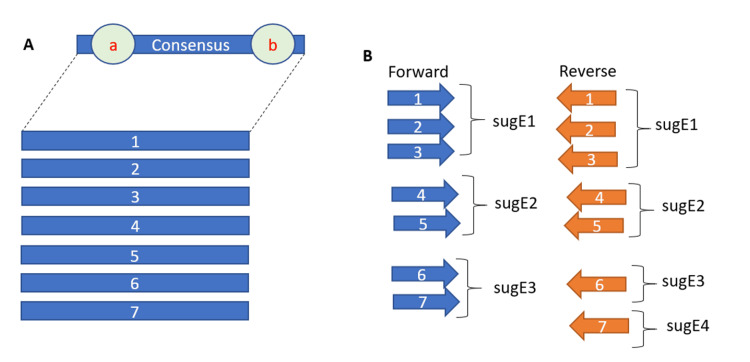
Schematic design of a universal primer mix. (A) Schematic shows how the multiple sequence alignment was made to yield targeting sites. (a) is where the forward primer was designed against, while (b) was the location where the reverse primer was designed. (B) schematic shows how submixes were designed from primers with the most nucleotide similarities. This design choice was done to prevent the overuse of degenerate nucleotides. (1) *A. molluscorum*, (2) *C. kluyveri*, (3) *E. coli*, (4) *K. pneumoniae*, (5) *P. fluorescens*, (6) *P. putida*, (7) *Sphingmonas*

Using the consensus sequence as a reference, individual sugE sequences were grouped into subsequences that had higher sequence identity than each other to minimize the use of degenerate bases (Figure 3). 

Using the subsequence approach improved the percent identity score. The result was three subsequences for the forward primers and four subsequences for the reverse primers, where sugE3-R and sugE4-R were kept individual due to significant sequence differences from the MSA, which did not allow them to be paired, resulting in four primer sets (Table 1). 

**Table 1:** Complete sequence of each forward and reverse primer for sugE and intended targets. Tm is the melting temperature of the primer set.

Primer pair	Primer sequence (forward/reverse)	T_m _(°C)
sugE1_F/sugE1_R	GTCCAGAYRGCRTAGGCGGT/TGGGCSRTYGGCCTSAAA	60
sugE2_F/sugE2_R	GTCCAGACCGCRTAAGCGGT/TGGGCTTTTTCGATGAAA	50
sugE3_F/sugE3_R	GTCCAGATKGYATAYGCMGT/TGGGCTGTAGGCTTGAAG	54
sugE3_F/sugE4_R	GTCCAGATKGYATAYGCMGT/TGGGCCTTGAGCCTCAAA	57

When designing universal primers, the introduction of a degenerate base increases the number of unique sequences in the primer set. For example, in sugE1-F, 3 degenerate nucleotides were used resulting in a mixture of eight primers. 

Within each primer set, each use of a generated nucleotide multiples the total combinations of primers by the number of nucleotides it encodes (two to four). For example, in sugE1-F, the use of three degenerate nucleotides resulted in a mixture of eight primers (Table 2).

**Table 2:** Degenerate primer combination mixes for sugE1_F

Primer	Sequence
sugE1-F (Degenerate Primer)	GTCCAGAYRGCRTAGGCGGT
Klebsiella (Target 1)	GTCCAGACGGCGTAGGCGGT
Pseudomonas Putida (Target 2)	GTCCAGATGGCATAGGCGGT
Spingomonas (Target 3)	GTCCAGATAGCGTAGGCGGT
All primer combinations (2×2×2)	GTCCAGACAGCATAGGCGGT
	GTCCAGACAGCGTAGGCGGT
	GTCCAGACGGCATAGGCGGT
	GTCCAGACGGCGTAGGCGGT
	GTCCAGATAGCATAGGCGGT
	GTCCAGATAGCGTAGGCGGT
	GTCCAGATGGCATAGGCGGT
	GTCCAGATGGCGTAGGCGGT

These primers were used for an in-silico genome screen to visualize unannotated gene targets. In all binding cases, only one primer (forward or reverse) annealed, which would result in no amplicons being formed in the in vitro amplifications (Table 3).

**Table 3:** In silico primer screening of the model community. Model community species were screened with Benchling by reducing stringency of binding parameters to three mismatches. Genes that were found to be bound by the primers were recorded.

Species	Annotation	Protein identity
B. subtilus	QU35_07305	Major facilitator transporter
B. subtilus	QU35_06870	Multidrug resistance protein
B. subtilus	QU35_17965	Fumarate hydratase
B. subtilus	QU35_19060	Exonuclease ABC subunit
B. subtilus	QU35_19235	Small multidrug-resistant protein
D. vulgaris	DvMF_3195	Histidine kinase
D. vulgaris	DvMF_0533	Cytochrome c oxidase
D. vulgaris	DvMF_0963	S-adenosyl methyltransferase
D. vulgaris	DvMF_1804	Alkyl hydroperoxide reductase
G. subterraneus	GSUB_04080	Hypothetical protein
G. subterraneus	GSUB_10675	Hypothetical protein
T. aminoaromactica	Tmz1t_2095	Heavy metal efflux pump

In most binding cases, primers annealed to genes that are predicted to encode a transporter.

In-vitro testing of primer sets

Gene blocks are synthetic pieces of DNA that act as a positive control for the PCR experiments. The sequences were designed to be identical to the primer set sequence. Hence, when amplified with PCR, these gene blocks would always generate an amplicon. The primer sets were verified by evaluating them on the positive control where an amplicon was confirmed. Increasing the concentration of the positive control template gave a more concentrated amplicon band when visualized by electrophoresis. 

**Figure 4:**
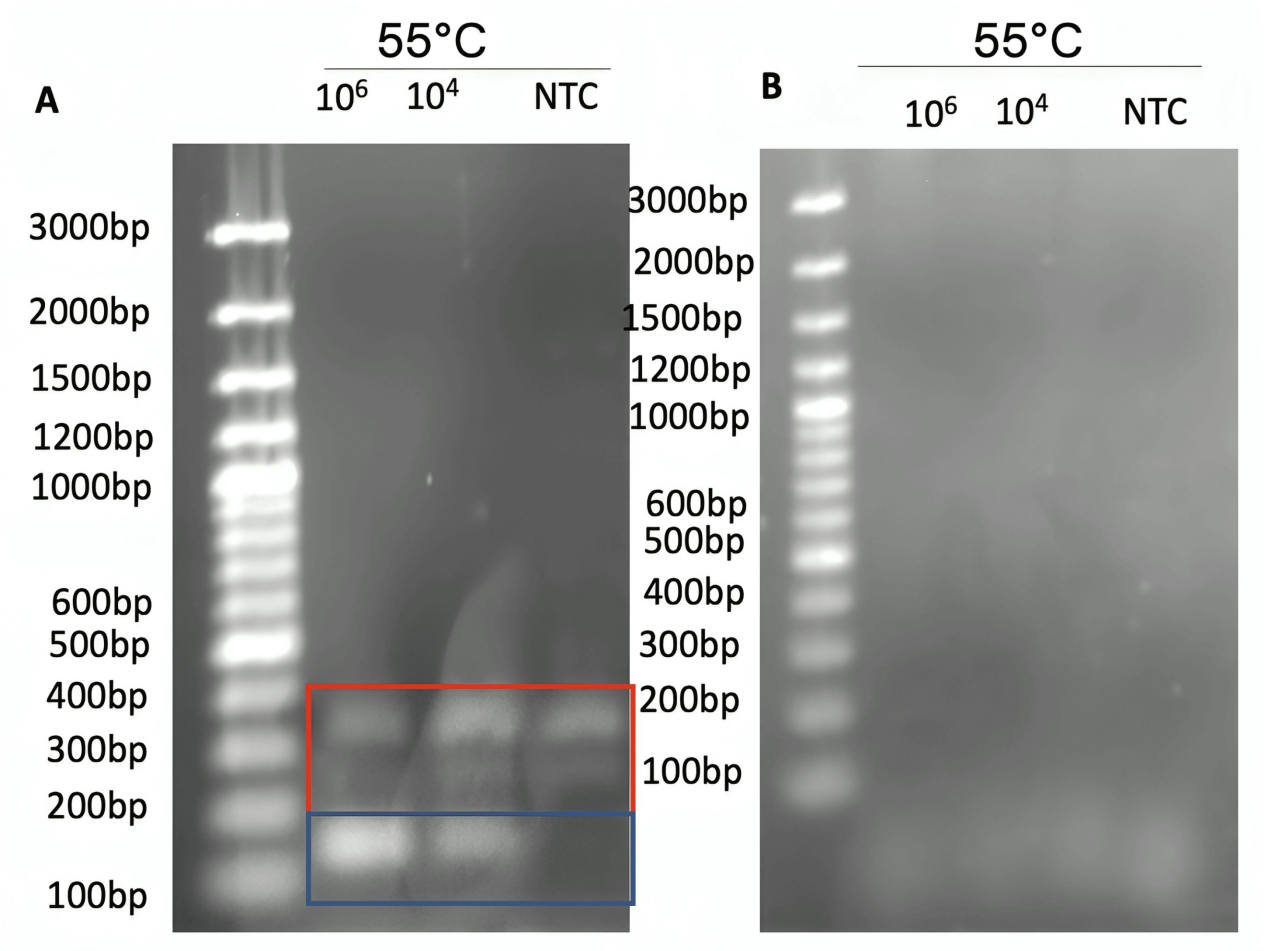
1.5% agarose gel of PCR amplification of positive control. (A) Agarose gel shows the possible successful amplification of the desired amplicon with undesired amplification products. (B) Agarose gel shows the replacement of contained primer mixture and the failure to obtain the same amplicon. NTC identifies the no template control, while the gene blocks are measured in gene copies/uL. Stock primer concentration was 10 mM and 1 uL of forward and reverse was used.

There were inconsistencies in primer sets received from different suppliers using the same PCR reagents and conditions. The primers received from IDT showed the appearance of DNA fragment brands regardless of the presence of a template at 350 bp and 250 bp (Figure 4A). It was believed that these were primer aggregates, but it was unable to be resolved through the temperature optimization through gradient PCR nor the replacement of reagents. The replacement of the primer set from the same supplier yielded no bands under the same conditions as the old primer set (Figure 4B).

**Figure 5:**
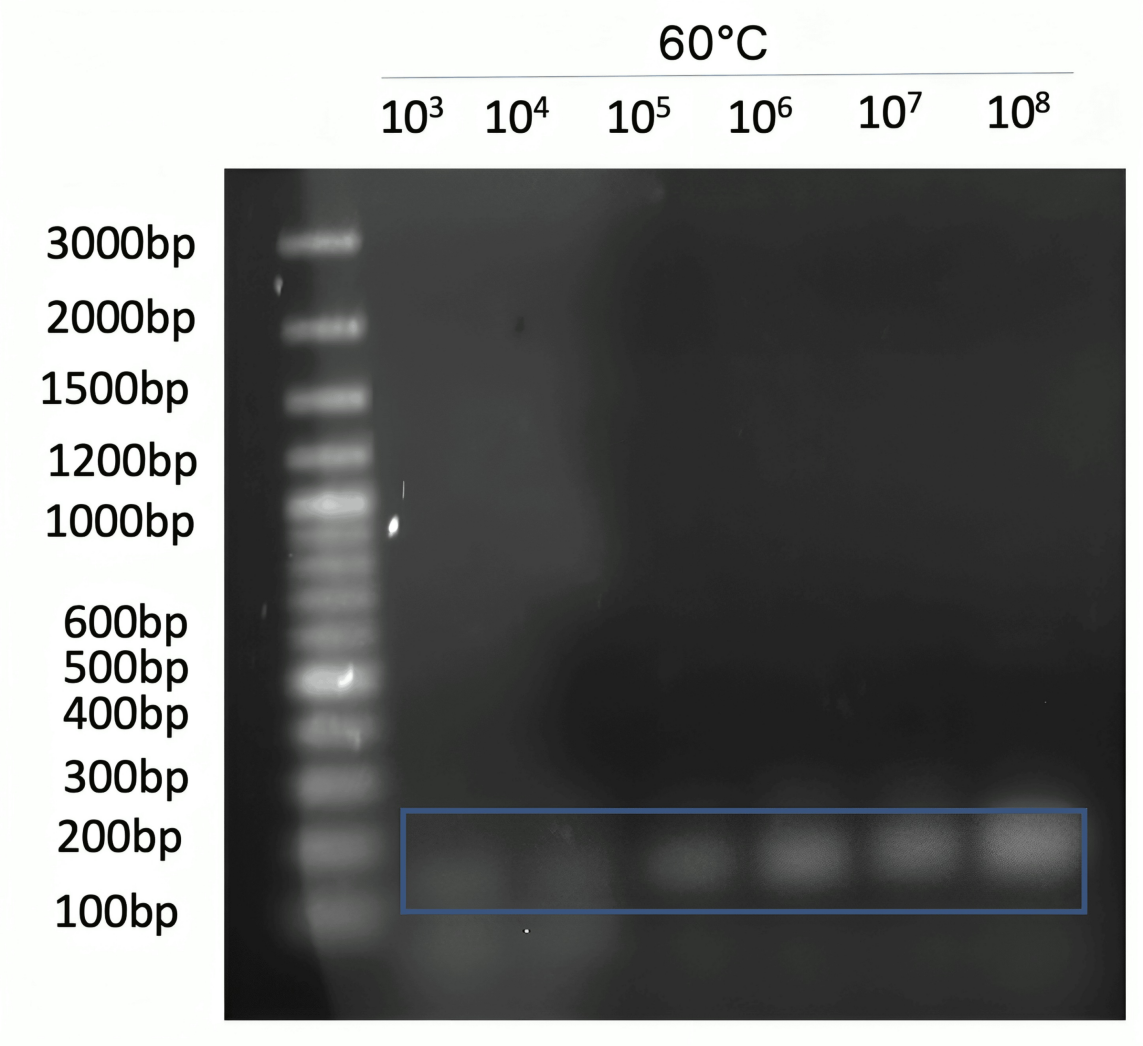
1.5% agarose gel of PCR amplification with new primers from a different supplier. Gene blocks were measured in copies/uL. Stock primer concentration was 10 mM and 1 uL of forward and reverse was used. The blue box shows the amplicon of interest.

This primer set was replaced by a different primer supplier and successfully produced an amplicon. A more concentrated amount of positive control template DNA yielded a darker band (Figure 5).

**Figure 6:**
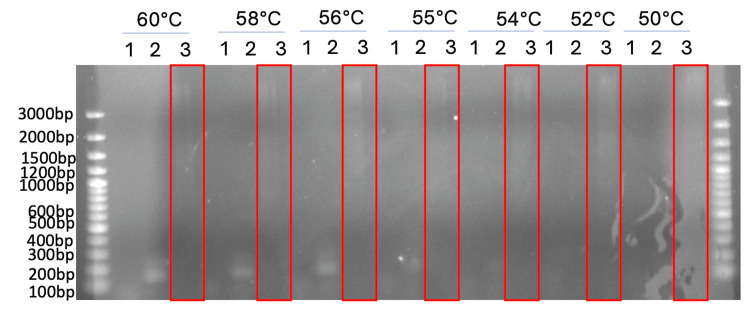
1.5% agarose gel of PCR amplification at different temperatures. (1) is the genomic DNA extracted from Pseudomonas putida, (2) is the synthetic gene block, (3) is the non-template control (NTC). Stock primer concentration was 10 mM and 1 uL of forward and reverse was used.

Adapting the working conditions to genomic DNA proved challenging, as the detection protocol that worked on the positive control could not be applied to the genomic DNA of one of the organisms used in the multiple sequence alignment (Figure 6).

## Discussion

This study describes a practical approach to designing and optimizing universal primers for detecting antibiotic resistance. As antibiotic resistance becomes more pressing, there is a growing need to detect antibiotic resistance genes before administering treatments.

Previous universal primer approaches have been used to target 16S rDNA genes. These primers have been successfully used for bacterial identification and metagenomic studies [25,26]. In line with this approach, specific antibiotic-resistance genes can be targeted for detection across bacterial species. One of the challenges faced in this study was the poor annotation of bacterial genomes. Most genes were annotated as a “hypothetical protein," which limited the scope of genes available to design against. The increasing availability of new methods may help characterize these hypothetical proteins and identify new gene targets [27]. However, unlike 16S rDNA, the antibiotic resistance genes of interest are accessory genes and have less fitness costs associated with mutations. This results in less conservation and increased difficulty in designing proper primer sequences. Coupled with their mobile nature, these genes can vary significantly across bacterial species, making it harder to design primers. These limitations were realized in the design process, where the MSA length, homologous regions, desired amplicon size, and primer size were all factors that limited the scope of the design. Compared to core genes like 16S rDNA, the gene sugE, which is part of the SMR family of transporters, is less conserved in sequence identity, making it harder to target with universal primers due to the increased use of degenerate nucleotides. Furthermore, the gene of interest is small at 300 bp, which limits the number of regions available to be targeted with primer designs. This suggests that it would be more practical to target larger genes. 

When designing primers, there is a trade-off between sensitivity and specificity. Considering most of the conserved regions within genes encode the transmembrane region that is important for the function of the protein, as more degenerate bases are used, unintended binding targets will increase raising the chances of false positive conclusions [28]. However, choosing to use fewer degenerate bases reduces the sensitivity of the primer set, which is out of the scope of the intended “universal” application of the primers to detect the gene of interest across a broad range of species. As the specific design choices of the primer appear to differ from a gene-by-gene basis, there is no clear guideline on the optimal number of degenerate bases to use, but this should be explored in future studies. Given that the primers are intended for simple diagnostic use, it is important to optimize and reduce these inefficiencies as much as possible.

As prokaryotic genome sequencing continues to grow, a shift to a more automatic annotation pipeline is necessary. As most automatic pipelines use homology methods to transfer information from a reference genome to a new sequence, this can propagate errors from poor annotations to new genomes. Furthermore, most genomes are annotated with “hypothetical proteins," which are genes encoding conserved proteins of unknown function or genes that encode a hypothetical protein [27]. Hence, the implementation of a primer-based annotation algorithm may be a good method to label genes accurately. This would provide a more comprehensive annotation with less error to public sequence databases. As most annotated genomes are well-studied models, a mechanism to detect antibiotic resistance genes is needed to understand the function of proteins in less well-studied organisms.

The in-silico tools used provided a valuable means of predicting biological phenomena, which is valuable in experimental design where running wet-lab experiments costs valuable time and resources. However, the transition to in vitro presents many barriers that cannot be accounted for in the bioinformatic analysis due to an increase in parameters. Even a simple variable like primer quality can vary between suppliers due to differences in synthesis protocols. Hence, more traditional tools need to be used to optimize the conditions. The formation of primer dimers, poor hybridization with complementary bases, and poor primer stability are all factors that make the protocol fragile, coupled with the competition between primer sets in a universal primer mixture can limit the quality of results obtained. The initial validation of primer sets showed by-product formation regardless of the presence or absence of a template in the reaction, which could not be resolved through the previously described optimization steps. Replacement with new primer sets from the same and an alternate supplier did not produce the same banding phenomenon that was observed, raising the need to consider primer supplier quality, which could be a source of false-positive or negative results when running PCR-based protocols. The lack of banding in both template and no template controls indicates no annealing phenomenon and hence no amplification product. 

This study showed the importance of optimization processes to improve sensitivity with gradient and matrix PCR to determine the optimal temperature and primer concentrations for wet-lab amplifications. Depending on the intended application of primers, annealing efficacy is important to obtain the intended results; hence, in vitro optimization steps are required. Although the primers worked on the positive synthetic control, this study was unable to obtain an amplification product using the previously mentioned optimization protocol with extracted genomic DNA of one of the organisms used to build the MSA. This was a result of poor primer annealing, as the 16S worked successfully on the same genomic DNA while the sugE primers failed to amplify a product.

Troubleshooting was not done in varying pH, ionic strength, or Taq because an in-house master mix was not used. Given the successful amplification of controls and 16S, this might indicate poor primer hybridization efficiency with genomic DNA, which could be addressed by experimenting with different polymerases. This would entail the use of lower fidelity polymerases with relaxed base pairing geometries, which would support the theory of the poor annealing of primers. Given this observation, primer design and binding should be revisited to investigate whether this is true.

This study showed the limitations of such approaches in multidrug resistance detection. Given the low identity score of the MSA for sugE, this method may prove difficult in community-wide detection and monitoring of accessory genes given the use of increased degenerate bases. Such cases increase false positivity through nonspecific binding and amplification or false negativity through incorrect annealing from increased competition from primer mixes. Ideally, working primers can detect the presence or absence of multidrug resistance in an environmental sample of bacteria without significant noise from nonspecific annealing.

## Conclusions

This study provides guidelines for a primer design strategy for detecting genes. Using the sugE gene from the SMR family as an example, universal primers were designed and tested to detect the gene across various microbial species. Previous studies have reported primers that detect the 16S rRNA gene. This study reports the challenges and complexity of designing primers for MDREP accessory genes, which exhibit low sequence conservation and require a delicate balance between specificity and sensitivity. Furthermore, the findings highlight the importance of optimizing both in silico and in vitro parameters to ensure successful primer use, yet also reveal the limitations of current primer-based detection methods for non-conserved genes in microbial communities. Future efforts should be directed toward refining the primer design process, exploring alternative polymerases, and further optimizing PCR conditions to minimize nonspecific binding and improve the accuracy of detecting resistance genes. Advancements in these areas are essential for enhancing diagnostic capabilities and addressing the growing challenge of antibiotic resistance in clinical environments.
